# Supplementary material for: The Dual Prey-Inactivation Strategy of Spiders—In-Depth Venomic Analysis of Cupiennius salei
Source: Toxins (Basel). 2019 Mar 19;11(3):167. doi: 10.3390/toxins11030167 (PMC6468893; doi:10.3390/toxins11030167)
Supplement: Supplementary file 1 [file toxins-11-00167-s001.zip › Supplementary Fig S1.1-S1.19/Supplementary Fig S1.11.pdf]

Supplementary Fig S1.11

CLUSTAL O(1.2.4) multiple sequence alignment: Angiotensin-converting enzymes

```
ACE_1_CUPSA                   ----MLGWAILHLASVLFASTVDSKGT-AVLRYASGTNDDYDAALEFLQENDALTGTIAN
ACE_2_CUPSA                   ----MLGWAILHLASVLFASTVDSKGT-AVLRYASGTNDDYDAALEFLQENDALTGTIAN
ACE_3_CUPSA                   ----MLGWAILHLTSVLFASTVDSKGT-AVLRYASGTNDDYDAALEFLQENDALTGTIAN
ACE_4_CUPSA                   ----MLGWAILHLTSVLFASTVDSKGT-AVLRYASGTNDDYDAALEFLQENDALTGTIAN
ACE_VIRFA                     ----MLATAILQVVSLLLAQTVNCREK-PIQKYTSGSNNDYDAAELFLWENDALTTIMAN
ACE_ALOMA                     ---TMLAWALLHLASVLLA---DGKGA-PIQRYASGNNADYDAAKNFLWENDVATGKIAN
tr_A0A087UCM7_STEMI           MLRMHLGTFLIPLLFLLFDSTVEGAGISLNEKYISGDNKNETAAIQFLQQHDIRLRKMLN
gi_XP_015910516.1_PARTE       ----MVSVFFLLQVVCLAASVSAACLN-KQDKYVSGDNTDLAAALRFLHDNDRLSEKITN
gi_KR068494_TITSE             ---MVL-----SLLFLLV--ITAYRLDVSGEKYISGDNRDEIAATQYLSAHNRILAEKCN
tr_Q9NDS8_BOMMO               MLKVGGGAVLIAAIVAVFI--VATQGR------DPDLEAREHEAREYMLHLDKATGLRKN
                                              :                  . :     *  ::   :       *

ACE_1_CUPSA                   KGAIAAWNYASNLTEENKKEMLKSQAEEAKFVKETWKNATSFAWKDFKERNETIYRWFKK
ACE_2_CUPSA                   KGAIAAWNYASNLTEENKKEMLKSQAEEAKFVKETWKNATSFAWKDFKERNETIYRWFKK
ACE_3_CUPSA                   KGAIAAWNYASNLTEENKKEMLKSQAEEAKFVKETWKNATSFAWKDFKERNETIYRWFKK
ACE_4_CUPSA                   KGAIAAWNYASNLTEENKKEMLKSQAEEAKFVKETWKNATSFAWKDFKERNETIYRWFKK
ACE_VIRFA                     KASIAQWNYASNLTEENKKAMLENQIEEGKFALESWKNATSFAWKDFKGKNETVYRWFKH
ACE_ALOMA                     KGAIAAWNYASNLTEHNKKVMLESQAEEAKFVKETWKNATSFAWKEFKDKDAFTYRWFKK
tr_A0A087UCM7_STEMI           KQTHASWNYASNLTEANKKNMLKVELQAAEFTKESWKNATSFAWKDFRTKNETIYRWFKK
gi_XP_015910516.1_PARTE       KGVIAQWNYQSNLTEHNKKEFLRMEEEMAKFAKETWKNATSFAWKNFKGKNETAYRWFKA
gi_KR068494_TITSE             IIVQAEWNYSTNITDENKQKLLEESSKYAEFQKEAWQNITSFAWKDFKD--PLIRRWFKK
tr_Q9NDS8_BOMMO               RASLAEWEYTSNITKENEEKSIQTHLELSRQEKAAWEETKMYGWQDFQD--FTLRRMFKK
                                  * *:* :*:*. *::  :.   : ..    :*:: . :.*::*:       * **

ACE_1_CUPSA                   LAKLGISALPEDQLDSFNSLVADMQDIYSKAKVCAKDSP-ADKPCELSLEPELSEIFKKS
ACE_2_CUPSA                   LAKLGISALPEDQLDSFNSLVADMQDIYSKAKVCAKDSP-ADKPCELSLEPELSEIFKKS
ACE_3_CUPSA                   LAKLGISALPEDQLDSFNSLVADMQDIYSKAKVCAKDSP-ADKPCELSLEPELSEIFKKS
ACE_4_CUPSA                   LAKLGISALPEDQLDSFNSLVADMQDIYSKAKVCAKDSP-ADKPCELSLEPELSEIFKKS
ACE_VIRFA                     LSVLGIAALPEDKLNTFNKLVADMQDTYAKAKVCSKDSP-ADKPCELSLEPELTEILKKS
ACE_ALOMA                     LTKLGTAALPEDQLEKFNTLVADMQDIYSKAKVCSKDSP-ADKPCDLSLEPELTEILKGT
tr_A0A087UCM7_STEMI           LSVLGYSALPPEDFKELSEIVADMQDIYSRAKICSYKSL-SKKDCNLSLEPELTEILATS
gi_XP_015910516.1_PARTE       LSVLGSAALPENKFNELGNINADMQDVYAKAKVCSYMSS-LLDPCTLSIEPELTEIMKES
gi_KR068494_TITSE             LSILGKAALPNDQLKELDELIADMKNIYSTSKVCPFNSKETDENCNLSLEPELTEILTKS
tr_Q9NDS8_BOMMO               YSQLGVAALPDDKFQALMRTVSGMESNYATAKICSYKNE---SKCDLSLEPEITEIFSTS
                               : ** :*** :.:. :    :.*:. *: :*:*   .    . * **:***::**:  :

ACE_1_CUPSA                   RDYNELKHVWTDWRKITGRKMKSEFLKYVRLSNEAALLNGFKDAGEMWREGYESETFADD
ACE_2_CUPSA                   RDYNELKHVWTDWRKITGRKMKSEFLKYVRLSNEAALLNGFKDAGEMWREGYESETFADD
ACE_3_CUPSA                   RDYNELKHVWTDWRKITGRKMKSEFLKYVRLSNEAALLNGFKDAGEMWREGYESETFADD
ACE_4_CUPSA                   RDYNELKHVWTDWRKITGRKMKSEFLKYVRLSNEAALLNGFKDAGEMWREGYESETFADD
ACE_VIRFA                     RDYEELKQVWNDWRAVSGKKVKPQFMEYVKLSNEAAKLNGFNDAGEMWRDRYETETFQED
ACE_ALOMA                     RDYNELKRVWADWRKVTGRKMREQFLEYVKLSNEAAELNGFKDAGEMWRDNYESDTFEKD
tr_A0A087UCM7_STEMI           TDYDELKHVWAGWRNATGKKMKDKYVKYVKLSNKASQLNGFSDTGMLWREVYESDDFEED
gi_XP_015910516.1_PARTE       TNYYELKHVWAQWRDETGKKMKNKFLRYVELSNEAACKNGFSDAGEMWREEFETDNITEE
gi_KR068494_TITSE             RNYDELAYIWKAWRDVSGKRVKDKYIRFIELSNKAAELNGFRDTGELWRERYESDTFQQE
tr_Q9NDS8_BOMMO               QDPEELKHAWVEWHNAAGATAKKNFTDYVNLYNEAAKLNGFDNVAEWWQSEYEVPDFEEQ
                               :  **   *  *:  :*   : ::  ::.* *:*:  *** :..  *:. :*   : .:

ACE_1_CUPSA                   VEALWKEIEPFYKQLHAFVRKRLIERYPDCGIKPDGPIPAHLLGNMWAQSWGNIFDIVKP
ACE_2_CUPSA                   VEALWKEIEPFYKQLHAFVRKRLIERYPDCGIKPDGPIPAHLLGNMWAQSWGNIFDIVKP
ACE_3_CUPSA                   VEALWKEIEPFYKQLHAFVRKRLIERYPDCGIKPDGPIPAHLLGNMWAQSWGNIFDIVKP
ACE_4_CUPSA                   VEALWKEIEPFYKQLHAFVRKRLIERYPDCGIKPDGPIPAHLLGNMWAQSWGNIFDIVKP
ACE_VIRFA                     IEELWKVVEPFYKQLHAFVRRRLIQQYPDKEILPNGPIPAHLFGNMWSQTWDNIYSIVKP
ACE_ALOMA                     VEDLWKVIEPFYRQLHAFVRRRLIQQYPDSGIQQDGPIPAHLLGNMWAQSWGNIFDIVKP
tr_A0A087UCM7_STEMI           IEALFQKIKPFYKHMHAYVRRKLIQRYPKHGIKPDGPIPAHLLGNMWAQTWGNILPEVMP
gi_XP_015910516.1_PARTE       FDQLWETIKPFYEQLHAYVRRKLIKEYTDIHIKPDGPIPAHLLGNMWAQTWGNIYRFVAP
gi_KR068494_TITSE             IEQLWQQIRPLYEQLHAYVRRKLINIYGSDKIRHDGPIPAHILGNMWAQDWSGILQDITP
tr_Q9NDS8_BOMMO               LAKLWEDVKPLYQQLHAYVRKRLRDKYGDKVVSARGPIPAHLLGNMWAQTWNNIESFTRP
                              .  *:: :.*:*.::**:**::* . * .  :   ******::****:* *..*     *
```

```
ACE_1_CUPSA                  IKEKQFLDVSDRMRERKMTALDMFKMSEEFFTSLGLKPMTQEFWNRSIIEKPKDREIVCH
ACE_2_CUPSA                  IKEKQFLDVSDRMRERKMTAIDMFKMSEEFFTSLGLKPMTQEFWNRSIIEKPKDREIVCH
ACE_3_CUPSA                  IKEKQFLDVSDRMRERKMTALDMFKMSEEFFTSLGLKPMTQEFWNRSIIEKPKDREIVCH
ACE_4_CUPSA                  IKEKQFLDVSDRMRERKMTAIDMFKMSEEFFTSLGLKPMTQEFWNRSIIEKPKDREIVCH
ACE_VIRFA                    FPNKEFVDVSKRMIAKKMTPLEMVKLSEEFFMSLGLKPMTPEFWNRSIFEKPKNRTMVCH
ACE_ALOMA                    YKQKAYVDVSNKMKEQKMTALDMFKMSEEFFTSLGLKPMTEEFWNRSIIEKPKDREIICH
tr_A0A087UCM7_STEMI          YPKKKELDITSVMKEKKMKIIDMFTMSDEFFASVGLKKMTSEFWNNSIIEKPRDREMVCH
gi_XP_015910516.1_PARTE      YPKKKAVDVTDTMLEKKLNARDMFEVSEEFFTSLGLIPMTPTFWKKSIIEKPTDREMVCH
gi_KR068494_TITSE            YPEKPSVDITPKMIAKNMSALEIFKISEEFFTSVGLKAMTDEFWERSIIEKPKDREIVCH
tr_Q9NDS8_BOMMO              YPDKKEIDVTQAMRDQNYTPMKMFQMSDEFFRSLNLTAMPEKFWKNSIIEKPTDREIVCH
                               .*  :*::  *  :: .  .:. :*:*** *:.*  *   **:.**:*** :* ::**

ACE_1_CUPSA                  ASAWDFSDGKDFRIKMCTRINMEDLITVHHEMGHIQYDQQYAHQPAVFRGGANPGFHEAV
ACE_2_CUPSA                  ASAWDFSDGKDFRIKMCTRINMEDLITVHHEMGHIQYDQQYAHQPAVFRGGANPGFHEAV
ACE_3_CUPSA                  ASAWDFSDGKDFRIKMCTRINMEDLITVHHEMGHIQYDQQYAHQPAVFRGGANPGFHEAV
ACE_4_CUPSA                  ASAWDFSDGKDFRIKMCTRINMEDLITVHHEMGHIQYDQQYAHQPAVFRGGANPGFHEAV
ACE_VIRFA                    ASAWDFSDGKDFRIKMCTRVDMEDLITVHHEMGHIQYYQQYDHQPAVFRQGANPGFHEAV
ACE_ALOMA                    ASAWDFSDGKDFRIKMCTRINMEDLITVHHEMGHIQYDQQYAHHPAVFRSGANPGFHEAV
tr_A0A087UCM7_STEMI          ASAWDFYDGKDVRIKMCTRLNMNDFKTVHHEMGHIEYFLQYAHQPTVFRRGANPGFHEAI
gi_XP_015910516.1_PARTE      ASAWDFYNDGDVRIKMCTRIDMEDLITVHHEMGHVEYFLQYKVQPVVFRNGANSGFHEAI
gi_KR068494_TITSE            ASAWDFCDGKDFRIKQCTSINMEDFIVTHHEMGHIQYYQQYAHQPCVFREGANPGFHEAV
tr_Q9NDS8_BOMMO              ASAWDFFDGEDFRIKQCTTVDYEYFQTTHHEMGHIQYYLQYRDQPVVFRDGANQGFHEAV
                             ****** :. *.*** ** :: : : ..******::*  **  :* *** *** *****:

ACE_1_CUPSA                  GDVLALSVASSTHLKKIGLLDDIHEDEGMEINGLMKTALGKVAFLPFGYLIDSWRWKVFS
ACE_2_CUPSA                  GDVLALSVASSTHLKKIGLLDDIHEDEGMEINGLMKTALGKVAFLPFGYLIDSWRWKVFS
ACE_3_CUPSA                  GDVLALSVASSTHLKKIGLLDDIHEDEGMEINGLMKTALGKVAFLPFGYLIDSWRWKVFS
ACE_4_CUPSA                  GDVLALSVASSTHLKKIGLLDDIHEDEGMEINGLMKTALGKVAFLPFGYLIDSWRWKVFS
ACE_VIRFA                    GDVLALSVATTNHLKKIGLLDARDEDNETEINSLMITALKKVAFLPFGYLVDSWRWKVFS
ACE_ALOMA                    GDVLALSVATSSHLKKVGLMSDSDEDEEMEINGLMKTALDKVASLPVGYLIDSWRWKVFS
tr_A0A087UCM7_STEMI          GDVMALSVSTPQHWVKVGLVDDVEEDKEADINYLMGVALNKVVFLPFAYVMDNWRWKLFS
gi_XP_015910516.1_PARTE      GDVLALSVATPDHMKKIGLLKEVNHDPEAEINNLLKTALDKIAFVPFGFLIDAWRWRVFD
gi_KR068494_TITSE            GDCLALSTSTPKHLQTIGLLDHIAEDKKGDVNFLFSTALSKLAFLPFGYLIDLWRWGIFS
tr_Q9NDS8_BOMMO              GDTIALSVSSPKHLRRVGLATGDAEDEQTEINQLYKMGIDKIAFLPFAYTLDLFRYGVFR
                             ** :***.::  *   :**     .*   ::* *   .: *:. :*..: :* :*: :*

ACE_1_CUPSA                  GEFSDDQLNSKWWEQRLKYQGLCPPVRRTDEDLDAAAKYHVIADVPYIRYFVSHIIQFQF
ACE_2_CUPSA                  GEFSDDQLNSKWWEQRLKYQGLCPPVRRTDEDLDAAAKYHVIADVPYIRYFVSHIIQFQF
ACE_3_CUPSA                  GEFSDDQLNSKWWEQRLKYQGLCPPVRRTDEDLDAAAKYHVIADVPYIRYFVSHIIQFQF
ACE_4_CUPSA                  GEFSDDQLNSKWWEQRLKYQGLCPPVRRTDEDLDAAAKYHVIADVPYIRYFVSHIIQFQF
ACE_VIRFA                    GEFPEEELNSKWWEQRLKYQGICPPVKRTNDDLDAASKYHVIADVPYIRYFVSTIIQFQF
ACE_ALOMA                    GEFSEDELNSKWWEQRLKYQGLCPPVRRTNDDLDAAAEYHVIADVPYIRYFVSHIIQFQF
tr_A0A087UCM7_STEMI          GEIKEDEMNSEWWNMRLKYQGLCPPVKRTNEDLDAACKYHTISDVKYIRYFVAHIIQFQF
gi_XP_015910516.1_PARTE      GSVKTNQLNSAWWNLRLKYQGVCPPVLRTEKDLDAAAKYHVIADVPYIRYFVSFVIQFQF
gi_KR068494_TITSE            GDIKSTELNTKWWELRLKYQGICPPVERTENDFDPGSKYHVPGNTPYVRYFVSHVIQFQF
tr_Q9NDS8_BOMMO              RKTLPEDYNCHYWKLREQLQGVEPPVNRTEDDFDAAAKYHVSSNVEYARYYVSFIIQFQF
                              .    : *  :*: * : **: *** **:.*:* ..:**. .:. * **:*: :*****

ACE_1_CUPSA                  HKALCEAAGHS------GPLHQCDIYQNKEAGKLLSDTLSLGSSVHWSEAMKVITRGKTD
ACE_2_CUPSA                  HKALCEAAGHS------GPLHQCDIYQNKEAGKLLSDTLSLGSSVHWSEAMKVITRGKTD
ACE_3_CUPSA                  HKALCEAAGHS------GPLHQCDIYQNKEAGKLLSDTLSLGSSVHWSEAMKVITRGKTD
ACE_4_CUPSA                  HKALCEAAGHS------GPLHQCDIYQNKEAGKLLSDTLSLGSSVHWSEAMKVITRGKTD
ACE_VIRFA                    HKALCDVAGYK------GPLHECDIYQNKDAGKLLSDTLRLGSSLHWTEAMNIITRGKTN
ACE_ALOMA                    HKALCEAAGHE------GPLHQCDIYQNKEAGKLLSDTLSLGSSVHWSEAMNVITRNTTR
tr_A0A087UCM7_STEMI          HKALCDAAGHE------GPLHKCDIYRSENAGKLLRDMMRLGSSVHWEKAMQIITNGKTY
gi_XP_015910516.1_PARTE      HKALCDEAGFK------GPLHKCDIYQNKNAGKLLSDTLKLGSSVSWREAMKVITKGKTD
gi_KR068494_TITSE            HKSLCEAAGHV------GPLHKCDIYKSKDAGKVLSQMLELGSSEIWNEAMNIMTGGVTN
tr_Q9NDS8_BOMMO              HRGVCQLAGEHAAGDPNKKLVDCDIYQSVAAGNALANMLKMGSSKPWPDAMEALTGQR--
                             *:.:*: **          * .****:.  **: * : : :***  * .**: :*

ACE_1_CUPSA                  KMDAQPMIEYFEPLMKWLKEEN--EGETLGWKSIDPMMCPENHNIKHF------------
ACE_2_CUPSA                  KMDAQPMIEYFEPLMKWLKEEN--EGETLGWKSIDPMMCPENHNIKHF------------
ACE_3_CUPSA                  KMDAQPMIEYFEPLMKWLKEEN--EGETLGWKSIDPMMCPENHNIKHF------------
```

```
ACE_4_CUPSA                KMDAQPMIEYFEPLMKWLKEEN--EGETLGWKSIDPMMCPENHNIKHF------------
ACE_VIRFA                  KMDAEPLIEYFEPLLRWLKKQN--EGETVGWKSIDPMICPQNPFIKHY------------
ACE_ALOMA                  KMDAQPLMEYFEPLIKWLQKQN--EGESLGWKSIDPMMCPENHNIK--------------
tr_A0A087UCM7_STEMI        KMDAKPLLEYFDPLIKWLHEKN--KNETVGWKSSSPMECPQ-------------------
gi_XP_015910516.1_PARTE    KMDASAILEYFKPLYDWLKDQN--KNEYIGWKSKDPMMCP--------------------
gi_KR068494_TITSE          KMDAGPMFEYFEPLYQWLKEEN--KGEVIGWKSGNPLFCP--------------------
tr_Q9NDS8_BOMMO            EMKADGLLEYFRPLHDWLRAENQRTGEHIGWEPTNMEYCTPSQLSELNVKEPSSSPATQQ
                           :*.*  ::*** **  **: :*   .* :**:  .   *

ACE_1_CUPSA                ---
ACE_2_CUPSA                ---
ACE_3_CUPSA                ---
ACE_4_CUPSA                ---
ACE_VIRFA                  ---
ACE_ALOMA                  ---
tr_A0A087UCM7_STEMI        ---
gi_XP_015910516.1_PARTE    ---
gi_KR068494_TITSE          ---
tr_Q9NDS8_BOMMO            SDS
```

Percent Identity Matrix - created by Clustal2.1

1: ACE_1_CUPSA	100.00	99.84	99.84	99.68	74.61	83.78	63.80	64.86	59.16	45.57
2: ACE_2_CUPSA	99.84	100.00	99.68	99.84	74.45	83.62	63.96	64.86	59.00	45.57
3: ACE_3_CUPSA	99.84	99.68	100.00	99.84	74.61	83.62	63.80	64.86	59.16	45.57
4: ACE_4_CUPSA	99.68	99.84	99.84	100.00	74.45	83.47	63.96	64.86	59.00	45.57
5: ACE_VIRFA	74.61	74.45	74.61	74.45	100.00	75.04	61.24	63.74	58.67	45.57
6: ACE_ALOMA	83.78	83.62	83.62	83.47	75.04	100.00	62.24	63.24	58.12	45.15
7: tr_A0A087UCM7_STEMI	63.80	63.96	63.80	63.96	61.24	62.24	100.00	61.34	58.48	45.95
8: gi_XP_015910516.1_PARTE	64.86	64.86	64.86	64.86	63.74	63.24	61.34	100.00	55.43	46.00
9: gi_KR068494_TITSE	59.16	59.00	59.16	59.00	58.67	58.12	58.48	55.43	100.00	45.81
10: tr_Q9NDS8_BOMMO	45.57	45.57	45.57	45.57	45.57	45.15	45.95	46.00	45.81	100.00

Proteomic coverage trypsin

>ACE_1_CUPSA angiotensin-converting enzyme isoform isoform 1 / 2 / 3 / 4
(I[315]L not differentiable)

MLGWAILHL**A** SVLFASTVDS KGTAVLR**YAS GTNDDYDAAL EFLQENDALT GTIANKGAIA AWNYASNLTE ENKKEMLKSQ**

AEEAKFVKET WKNATSFAWK DFKERNETIY R**WFKKLAKLG ISALPEDQLD SFNSLVADMQ DIYSKAKVCA KDSPADKPCE**

LSLEPELSEI FKKSRDYNEL KHVWTDWRKI TGR**KMKSEFL KYVRLSNEAA LLNGFKDAGE MWREGYESET FADDVEALWK**

EIEPFYKQLH AFVRKR**LIER YPDCGIKPDG PIPAHLLGNM WAQSWGNIFD IVKPIKEKQF LDVSDRMRER KMTALDMFKM**

SEEFFTSLGL KPMTQEFWNR SIIEKPKDRE IVCHASAWDF SDGKDFRIKM CTR**INMEDLI TVHHEMGHIQ YDQQYAHQPA**

VFRGGANPGF HEAVGDVLAL SVASSTHLKK IGLLDDIHED EGMEINGLMK TALGKVAFLP FGYLIDSWRW KVFSGEFSDD

QLNSKWWEQR LKYQGLCPPV RRTDEDLDAA AKYHVIADVP YIRYFVSHII QFQFHKALCE AAGHSGPLHQ CDIYQNKEAG

KLLSDTLSLG SSVHWSEAMK VITRGKTDKM DAQPMIEYFE PLMKWLKEEN EGETLGWKSI DPMMCPENHN IKHF

Proteomic coverage chymotrypsin

>ACE_1_CUPSA angiotensin-converting enzyme isoform 1 / 2 / 3 / 4
(I[315]L not differentiable)

MLGWAILHL**A** SVLFASTVDS **KGTAVLRYAS GTNDDYDAA**L EF**LQENDALT GTIANKGAIA AW**NYASNLTE ENKKEM**LKSQ**

AEEAKFVKET WKNATSFAWK DFKERNETIY RW**FKKLAKLG ISALPEDQLD SFNSLVADMQ DIYSKAKVCA KDSPADKPCE**

LSLEPELSEI FKKSRDYNEL KHVWTDWRKI TGRKMKSEFL KYVRLSNEAA LLNGFKDAGE MWREGYESET FADDVEALWK

EIEPFYKQLH AFVRKRL**IER YPDCGIKPDG PIPAHLLGNM WAQSWGNIFD IVKPIKEKQF L**DVSDRMRER **KMTALDMFKM**

SEEFFTSLGL KPMTQEFWNR SIIEKPKDRE IVCHASAWDF SDGKDFRIKM CTRINMEDLI TVHHEMGHIQ YDQQYAHQPA

VFRGGANPGF HEAVGDVLAL SVASSTHLKK IGLLDDIHED EGMEINGL**MK TALGKVAFLP F**GY**LIDSWRW** KVF**SGEFSDD**

QLNSKWWEQR LKYQGLCPPV RRTDEDLDAA AKYHVIADVP YIRYFVSHII QFQFHKALCE AAGHSGPLHQ CDIYQNKEAG

KLLSDTLSLG SSVHWSEAMK VITRGKTDKM DAQPMIEYFE PLMKWLKEEN EGETLGWKSI DPMMCPENHN IKHF

Supplementary Fig S1.11 Multiple sequence alignment of ACE from the venom glands of *C. salei* (ACE_1_CUPSA [MH766632]), (ACE_2_CUPSA [MH766633]), (ACE_3_CUPSA [MH766634]), (ACE_4_CUPSA [MH766635]), *A. marikovskyi* (ACE _ALOMA), *V. fasciatus* (ACE _VIRFA). *S. mimosarum* (tr_A0A087UCM7_STEMI), *P. tepidariorum* (gi_XP_015910516.1_PARTE), the scorpion *Tityus serrulatus* (gi_KR068494_TITSE), and the insect *Bombyx mori* (tr_Q9NDS8_BOMMO). Signal peptides are marked in black characters, cysteines in white with a black box, positively charged amino acids in pink, negatively charged amino acids in blue, hydrophobic amino acids in red and polar amino acids in green. Residues involved in Zn^{2+} binding are highlighted in yellow and active site residues in gray.
